# Adrenomedullin Mitigates Doxorubicin-Induced Nephrotoxicity in Rats: Role of Oxidative Stress, Inflammation, Apoptosis, and Pyroptosis

**DOI:** 10.3390/ijms232314570

**Published:** 2022-11-23

**Authors:** Rania Nagi Abd-Ellatif, Nahla Anas Nasef, Hemat El-Sayed El-Horany, Marwa Nagy Emam, Reham Lotfy Younis, Rehab E. Abo El Gheit, Walaa Elseady, Doaa A. Radwan, Yasser Mostafa Hafez, Ahmad Eissa, Alshimaa Aboalsoud, Rania H. Shalaby, Marwa Mohamed Atef

**Affiliations:** 1 Medical Biochemistry Department, Faculty of Medicine, Tanta University, Tanta 31527, Egypt; 2 Biochemistry Department, College of Medicine, Ha’il University, Ha’il 2440, Saudi Arabia; 3 Physiology Department, Faculty of Medicine, Tanta University, Tanta 31527, Egypt; 4 Anatomy and Embryology Department, Faculty of Medicine, Tanta University, Tanta 31527, Egypt; 5 Internal Medicine Department, Faculty of Medicine, Tanta University, Tanta 31527, Egypt; 6 Pharmacology Department, Faculty of Medicine, Tanta University, Tanta 31527, Egypt; 7 Dubai Medical College for Girls, Dubai 20170, United Arab Emirates

**Keywords:** nephrotoxicity, doxorubicin, adrenomedullin, pyroptosis, gasdermin

## Abstract

Doxorubicin (DOX) is an anticancer antibiotic which has various effects in human cancers. It is one of the commonly known causes of drug-induced nephrotoxicity, which results in acute renal injury. Adrenomedullin (ADM), a vasodilator peptide, is widely distributed in many tissues and has potent protective effects. Therefore, the current study aimed to examine the protective potential mechanisms of ADM against DOX-induced nephrotoxicity. A total of 28 male Wistar rats were randomized into four groups: control group, doxorubicin group (15 mg/kg single intraperitoneal injection of DOX), adrenomedullin + doxorubicin group (12 μg/kg/day intraperitoneal injection of ADM) 3 days prior to DOX injection and continuing for 14 days after the model was established, and adrenomedullin group. Kidney function biomarkers, oxidative stress markers, and inflammatory mediators (TNF-α, NLRP3, IL-1β, and IL-18) were assessed. The expressions of gasdermin D and ASC were assessed by real-time PCR. Furthermore, the abundances of caspase-1 (p20), Bcl-2, and Bax immunoreactivity were evaluated. ADM administration improved the biochemical parameters of DOX-induced nephrotoxicity, significantly reduced oxidative damage markers and inflammatory mediators, and suppressed both apoptosis and pyroptosis. These results were confirmed by the histopathological findings and revealed that ADM’s antioxidant, anti-inflammatory, anti-apoptotic, and anti-pyroptotic properties may have prospective applications in the amelioration of DOX-induced nephrotoxicity.

## 1. Introduction

Doxorubicin (DOX), also identified as adriamycin, is a well-known anticancer anthracycline antibiotic with a potent antitumor effect against a wide diversity of human tumors, including hematological malignancies and cervical, ovarian, and uterine cancers [1]. Unfortunately, DOX’s devasting toxic effects on many organs, including the heart, liver, testes, and kidneys limit its use [2]. Tubular atrophy along with increased glomerular capillary permeability are among the most common pathologies caused by DOX in rats’ kidneys [3], representing a condition of acute kidney injury (AKI). AKI is considered as a potentially fatal illness with a significant global health impact [4]. Accordingly, there has been a lot of interest in finding a medication that might prevent AKI.

Though the exact mechanism causing DOX-induced nephrotoxicity is still unidentified, reactive oxygen species (ROS) buildup may be one of the underlying mechanisms explaining this nephrotoxicity. This may be attributed to the DOX chemical structure which leads to ROS production, resulting in oxidative damage of numerous biological macromolecules and peroxidation of membrane lipids [5]. Additionally, DOX-induced oxidative stress stimulates TNF-α release, which activates several signaling pathways containing the nuclear factor kappa B (NF-κβ) inflammatory pathway [6]. In addition, DOX has a powerful apoptotic effect, where it mainly affects the mitochondrial pathway [7].

Pyroptosis, a recently identified form of programmed cell death, exists exclusively in dendritic cells and macrophages [8]. Moreover, it occurs in other organs, including the liver [9] and kidneys [10,11]. Pyroptosis is recognized by the rapid rupture of the plasma membrane, DNA damage, and the release of inflammatory cytokines [12,13]. This form of cell death is stimulated by pore-forming proteins, known as gasdermin, which are substrates of both caspase-1 and caspase-4/5/11 that generate N-terminal fragments [14,15]. The NOD-like receptor (NLR) and ASC together build up the multiprotein platform known as the inflammasome. NLRP3 is the best-characterized type of inflammasome [16]. Assemblage of NLRP3, ASC, and procaspase triggers NLRP3 inflammasome activation [17]. The active inflammasome then recruits and activates caspase-1, which is followed by the cleavage and subsequent activation of proinflammatory cytokines such as IL-1β and IL-18, resulting in pyroptosis [16].

Adrenomedullin (ADM), a biologically active endogenous peptide composed of 52 amino acids, has been separated from the pheochromocytoma of human tissues. However, it has been isolated from several tissues later on including lung, adrenal gland, heart, blood vessels, reproductive organs, and kidneys [18]. ADM has powerful antioxidant, anti-apoptotic, and anti-inflammatory properties, where it has been used to mitigate renal damage in numerous models of renal diseases such as ischemia reperfusion injury [19], mesangioproliferative glomerulonephritis, and unilateral ureteral obstruction [20]. Furthermore, ADM was previously reported to alleviate pyroptosis in Leydig cells [21]. Nevertheless, its effect on doxorubicin-induced nephrotoxicity has not yet been examined. The current study was intended to validate the renoprotective effect of ADM on doxorubicin-induced nephrotoxicity, highlighting its potential anti-pyroptotic effect.

## 2. Results

### 2.1. Effect on Body, Relative Kidney Weight, and Renal Function Parameters

**Figure 1:**
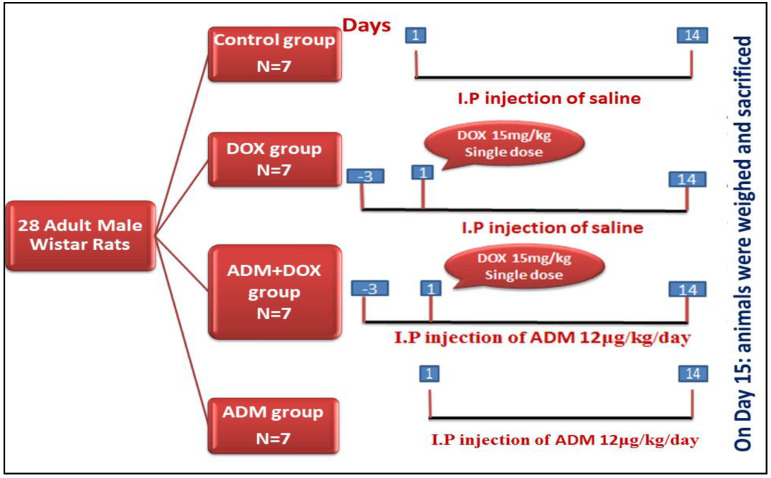
Simple schematic representation of the experimental protocol.

A single intraperitoneal injection of DOX was used to induce the renal injury model in rats. Rats were sacrificed on day 15 after 2 weeks of ADM treatment (Figure 1). The effect of DOX and ADM co-administration on the body weight, kidney weight, and renal functions is shown in Table 1. Compared to the control group, both DOX and DOX co-treated groups showed significant reduction in body weight (*p* < 0.05); however, a significant increase was noticed in the relative kidney weight.

There was a significant increase in kidney biochemical parameters (serum creatinine and BUN) (*p* < 0.05) in the DOX-induced group compared to the normal control group. Creatinine and BUN levels in the DOX co-treated group were significantly decreased (*p* < 0.05) compared to DOX-only treated group; this implies that ADM preserved the renal function.

Furthermore, the effect of DOX and ADM on creatinine clearance and urinary KIM-1 were investigated. The DOX-induced group showed a significant decrease in creatinine clearance; however, it showed a significant increase in KIM-1 level (*p* < 0.05) in comparison to both control groups. The treatment of DOX-induced rats with ADM significantly attenuated DOX-induced changes in urinary KIM-1 level and creatinine clearance. The previous results indicate that ADM may regenerate kidney function and tissue. ADM treatment alone did not show any significant effect.

### 2.2. Effect of ADM on Redox and Inflammatory Status

Biomarkers of oxidative stress (MDA, CAT, and GSH) were estimated in renal tissue homogenates of all groups (Table 2). DOX administration significantly increased tissue MDA level but decreased CAT activity and GSH level compared to the control group. Co-treatment with ADM increased GSH levels to statistically insignificant levels compared to the control group, but there was still a significant difference in MDA level and CAT activity compared to the control group. The DOX group showed a significant increase in 8-OHdG levels compared to the control group, while ADM was found to have a protective effect on the DOX-induced DNA damage. In addition, it limited the rise of 8-OHdG levels, as stated in Table 2. Concurrently, the level of TNF-α in renal tissues was significantly increased in DOX-induced rats compared to those in control rats (Table 2). ADM co-administration significantly diminished the increase in the levels of TNF-α as compared with the DOX-intoxicated rats (*p* < 0.05). ADM treatment alone did not show any significant effect.

### 2.3. Effect of ADM on Inflammasome Protein and Pyroptosis Markers in Renal Tissues

**Figure 2:**
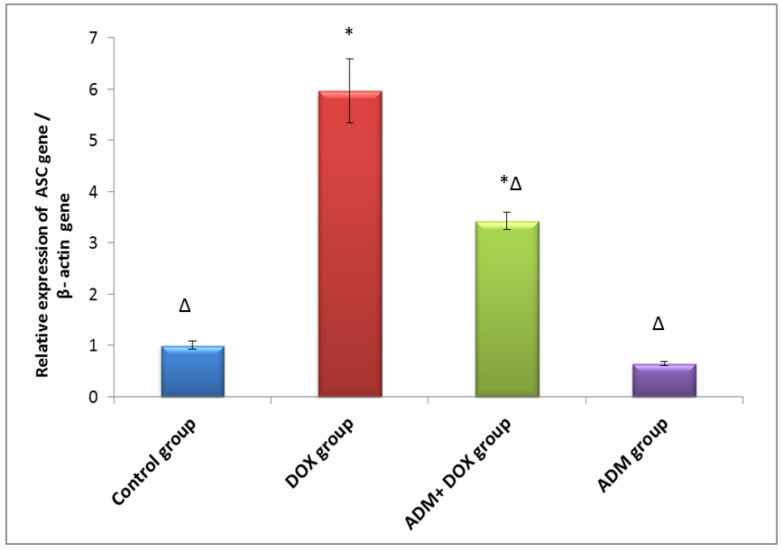
Effect of ADM treatment on renal *ASC* gene relative expression in all the studied groups. Data are expressed as mean ± standard deviation. ADM: adrenomedullin; *ASC*: apoptosis-associated speck-like protein containing a caspase recruitment domain; DOX: doxorubicin. Statistical analysis was carried out using one-way ANOVA with Tukey’s post hoc test, SPSS computer program. * Significant difference vs. control group (*p* < 0.05); Δ significant difference vs. DOX group (*p* < 0.05); n = 7 rats/each group.

**Figure 3:**
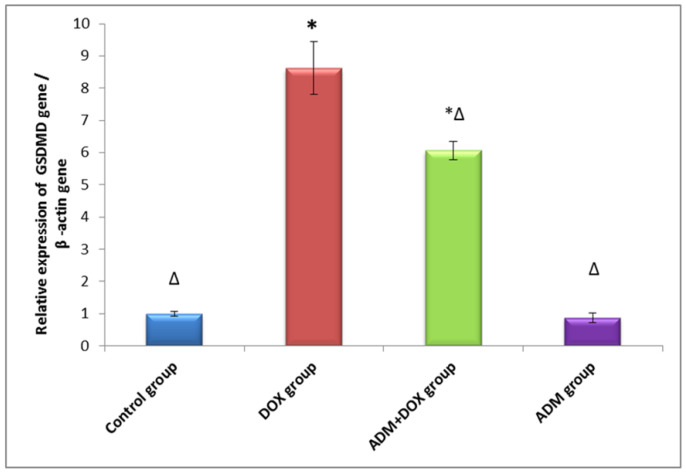
Effect of ADM treatment on renal *GSDMD* gene relative expression in all the studied groups. Data are expressed as mean ± standard deviation. ADM: adrenomedullin; DOX: doxorubicin; *GSDMD*: gasdermin D. Statistical analysis was carried out using one-way ANOVA with Tukey’s post hoc test, SPSS computer program. * Significant difference vs. control group (*p* < 0.05); Δ significant difference vs. DOX group (*p* < 0.05); n = 7 rats/each group.

**Figure 4:**
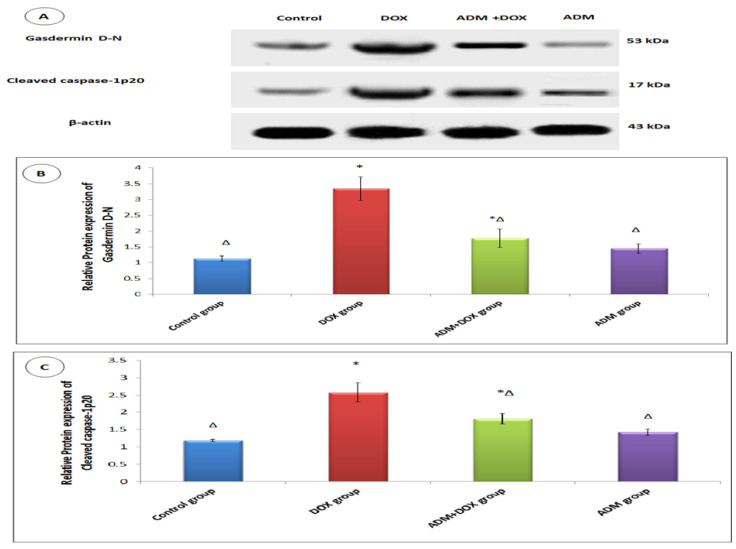
Effect of ADM treatment on renal GSDMD-N and cleaved caspase-1 p20 protein expression in all the studied groups (A-C). Western blot analysis of the relative expression of cleaved-GSDMD (B), active caspase-1 (p20) (C). Data are expressed as mean ± standard deviation. Statistical analysis was carried out using one-way ANOVA with Tukey’s post hoc test, SPSS computer program. ADM: adrenomedullin; DOX: doxorubicin. * Significant difference vs. control group (*p* < 0.05); Δ significant difference vs. DOX group (*p* < 0.05). Data for Western blot represent *n* = 3 biologically independent samples, each performed in duplicate.

The activation of the NLRP3 inflammasome was previously documented in renal injury induced by DOX. Therefore, we investigated the effects of ADM on DOX-induced inflammation. The DOX group had significantly higher levels of NLRP3, IL-18, and IL-1β compared to other groups (Table 3). Additionally, when compared to the control group, the DOX-only treated group showed upregulation of ASC and GSDMD gene expression (Figure 2 and Figure 3). Meanwhile, ADM co-treatment with DOX in group III markedly reduced NLRP3, IL-18, and IL-1β levels and downregulated ASC and GSDMD expression compared to the DOX-only treated group. These findings were further confirmed by a Western blot analysis, as shown in Figure 4A. A significant increase in the protein expression of GSDMD-N (Figure 4B) and caspase-1 p20 (Figure 4C) was observed in the DOX-only treated group compared to the control group. Moreover, ADM co-administration with DOX in group III diminished these proteins’ expression compared to the DOX-only treated group (Figure 4B,C). Collectively, these results imply that ADM improved DOX-induced renal injury by attenuating inflammation. ADM treatment alone did not show any significant effect.

### 2.4. DNA Ladder Assay

**Figure 5:**
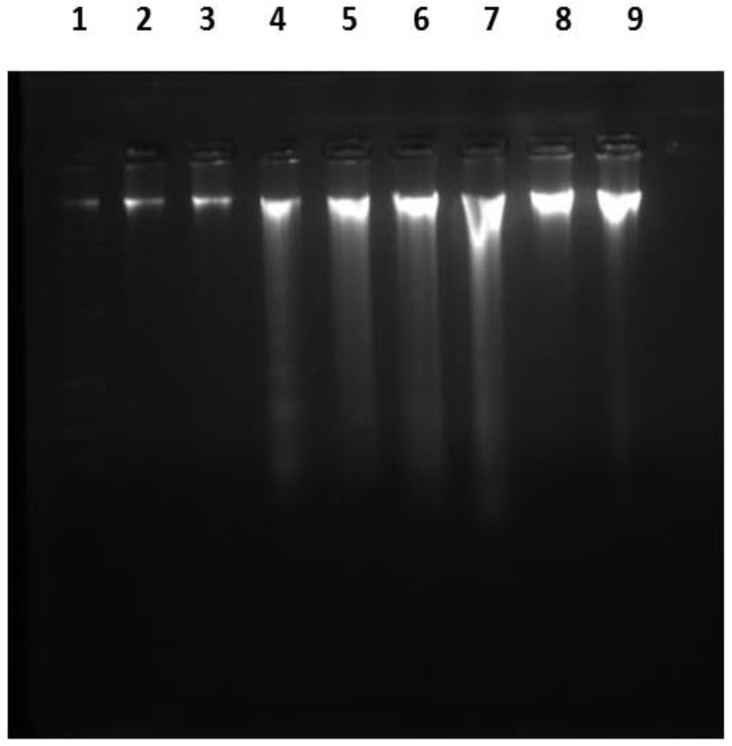
Electrophoretic pattern of DNA fragments. Lane 1: DNA marker (100–3000 bp); Lanes 2 and 3: control group; Lanes 4 and 5: DOX group; Lanes 6 and 7: ADM + DOX group; Lanes 8 and 9: ADM group.

In the current study, laddering assay demonstrated a normal intact band of DNA for samples taken from both control and ADM groups. Meanwhile, the DOX group showed a fragmented apoptotic DNA pattern. Concurrent treatment with ADM and DOX decreased the fragmentation of DNA, which protects against the harmful effect of DOX (Figure 5).

### 2.5. Effect of ADM on Renal Cortex Histological Features

**Figure 6:**
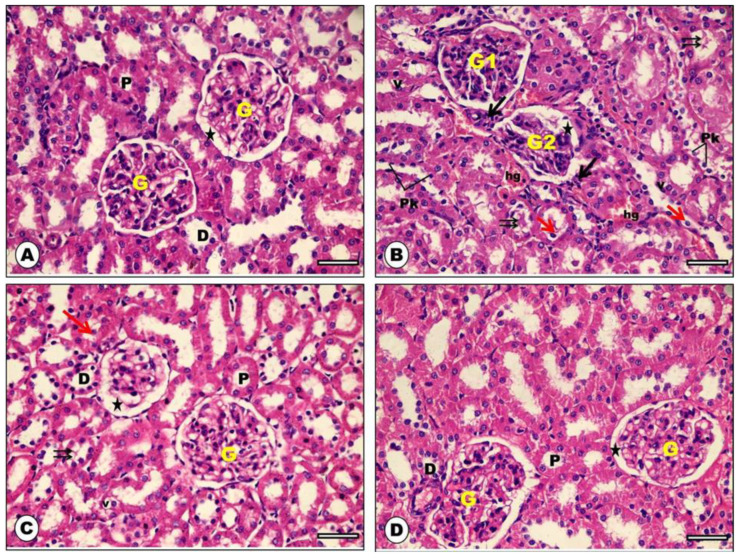
Photomicrograph of H&E-stained sections from rat renal cortex of the studied groups: (**A**) control group showed multiple glomeruli (G) surrounded with clear Bowman’s space (star). Proximal convoluted tubules (P) appeared with rounded basal vesicular nuclei and apical clear brush border. Distal convoluted tubules (D) appeared with central rounded nuclei. (**B**) DOX group showed disturbed organization of the renal cortex architecture. Some glomeruli appeared with congested capillaries (G1), the others appeared shrunken (G2) and surrounded with wide Bowman’s space (star) containing cellular debris. Some of epithelial lining of the renal tubules showed vacuolar degeneration (V) and cytoplasmic necrosis with pyknotic nuclei (Pk). Lumens of the tubules showed shedding of some epithelial cells (red arrows) and acidophilic cast (double arrows). Interstitial hemorrhage (hg) and focal mononuclear cellular infiltrate (black arrows) were also noticed. (**C**) ADM + DOX group showed a near-normal architecture of the renal cortex and normal glomeruli (G) surrounded with clear Bowman’s space (star). The proximal (P) and distal (D) tubules were normal to a great extent. However, degenerated vacuoles were found in a few of their lining epithelia (V). There were a few casts inside tubular cells and a few detached epithelial cells (red arrow) (**D**) ADM group showed well-organized architectural pattern of the renal cortex; multiple glomeruli (G) surrounded with clear Bowman’s space (star), normal proximal convoluted tubules (P), and distal convoluted tubules (D) (H&E × 400, scale bar = 50 μm).

The control and ADM groups showed sections of the renal cortex formed of multiple glomeruli (G) surrounded with clear Bowman’s space (star). Proximal convoluted tubules (P) appeared with rounded basal vesicular nuclei and an apical clear brush border. Distal convoluted tubules (D) appeared with central rounded nuclei (Figure 6A,D). The DOX group showed disturbed organization of the renal cortex architecture. Some glomeruli appeared with congested capillaries (G1), and the others appeared shrunken (G2) and surrounded with wide Bowman’s space (star) containing cellular debris. Some of the epithelial lining of renal tubules showed vacuolar degeneration (V) and cytoplasmic necrosis with pyknotic nuclei (Pk). Lumens of the tubules showed shedding of some epithelial cells and acidophilic cast. Interstitial hemorrhage (hg) and focal mononuclear cellular infiltrate were also noticed (Figure 6B). Concomitant administration of ADM and DOX showed a near-normal architecture of the renal cortex; normal glomeruli (G) were surrounded with clear Bowman’s space (star). The proximal (P) and distal (D) tubules were normal to a great extent. However, degenerated vacuoles were found in a few of their lining epithelia (V). There were a few casts inside tubular cells and a few detached epithelial cells (red arrow) (Figure 6C).

### 2.6. Effect of ADM on Bax Immunohistochemical Staining

**Figure 7:**
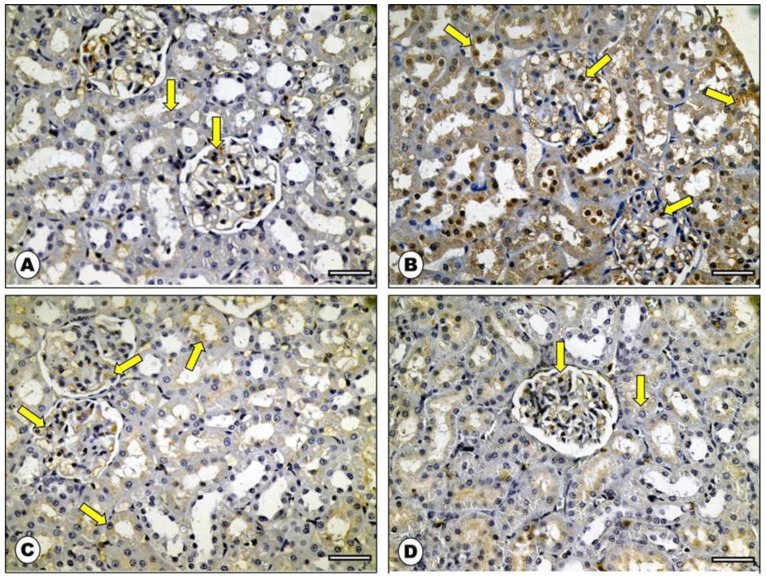
Photomicrograph of Bax immunohistochemical stained sections from rat renal cortex: (**A**) control group showed very faint positive brownish cytoplasmic immunoreactions (arrows). (**B**) DOX group showed strong positive reaction (arrows). (**C**) ADM + DOX group showed weak immunoreactivity of Bax in the cytoplasm of some of cortical cells (arrows). (**D**) ADM group depicted few cells with weak positive Bax immunoreaction (arrows) (Bax immunostaining: ×400, scale bar = 50 μm).

Immunostained sections from rats’ renal cortexes of both control and ADM groups showed very faint positive brownish cytoplasmic immunoreaction (Figure 7A,D). Meanwhile, sections from DOX group rats showed a strong positive reaction when compared with control rats (Figure 7B). On the other hand, sections from the DOX group co-treated with ADM showed weak immunoreactivity of Bax in the cytoplasm of some of the cortical cells (Figure 7C). In the DOX group, there was a significant increase in the morphometric analysis of the mean area percentage and color intensity of Bax-positive immunoreaction compared with both control groups, while the ADM co-treated group showed a non-significant difference in immunoreactivity compared to both control groups (Table 4).

### 2.7. Effect of ADM on Bcl-2 Immunohistochemical Staining

**Figure 8:**
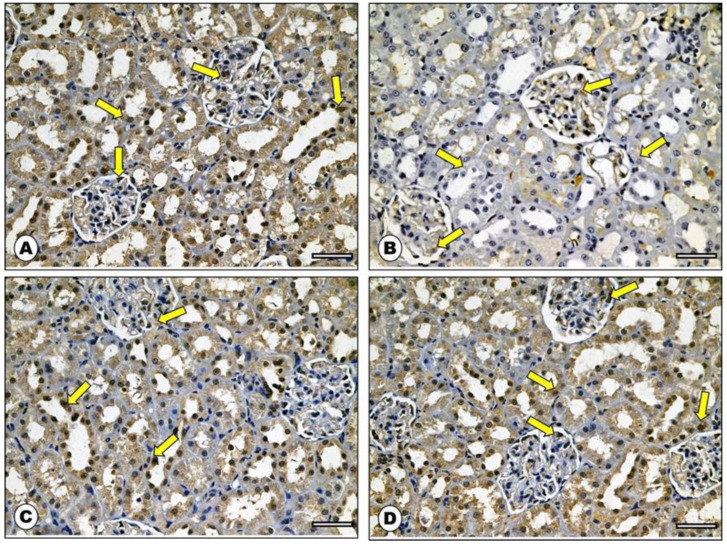
Photomicrograph of Bcl-2 immunohistochemical stained sections from rat renal cortex: (**A**) control group showed strong positive cytoplasmic Bcl-2 immunoreaction in the form of a brownish coloration (arrows). (**B**) DOX group showed weak reaction when compared with control rats (arrows). (**C**) ADM + DOX group showed moderate to marked reactivity of Bcl2 in the cytoplasm of the cortical cells (arrows). (**D**) ADM group depicted numerous cells with strong positive Bcl-2 immunoreaction (arrows) (Bcl-2 immunostaining: ×400, scale bar = 50 μm).

Immunostained sections from rats’ renal cortexes of both control and ADM groups showed strong positive cytoplasmic immunoreaction in the form of a brownish coloration (Figure 8A,D). Meanwhile, sections from the DOX group rats showed weak reaction in comparison to the control group (Figure 8B). However, the cytoplasm of cortical cells from the DOX group co-treated with ADM displayed moderate to marked reactivity of Bcl-2 (Figure 8C). The DOX intoxicated rats showed a significant decrease in the morphometric analysis of the mean area percentage and color intensity of Bcl-2-positive immunoreaction compared to both control groups, while there was a non-significant difference in Bcl-2 immunoreaction in the ADM co-treated group in comparison with both control groups (Table 4).

## 3. Discussion

Doxorubicin (DOX), an antitumor drug, is commonly used in the management of various solid and hematological malignancies [22]. Unfortunately, DOX has serious side effects, including nephrotoxicity [23]. Adrenomedullin (ADM), a vasodilator peptide, is widely distributed in many tissues and has a remarkable protective effect in many pathological conditions due to its anti-inflammatory, antioxidant, and anti-apoptotic effects [24].

The present work was planned to determine ADM efficacy to prevent chemotherapeutic-drug-induced renal damage, which could improve DOX’s antitumor effect. DOX-induced kidney damage was demonstrated by biochemical, histopathological, and immunohistochemical findings. However, it was discovered that injecting rats with ADM after DOX administration had a positive effect on these results, as it protected them from kidney damage.

The current study revealed a significant decrease in the body weight but an increase in the relative kidney weight in the DOX group. DOX-induced renal dysfunction was shown in the present study by increased serum creatinine and BUN levels, as well as decreased creatinine clearance in the DOX group compared to the control group. These results are in agreement with the results of previous research by Qiao et al. (2018) and Khames et al. (2019) [25,26]. In the DOX group, deteriorations in the renal function may be ascribed primarily to the overproduction of free radicals and reactive oxygen species (ROS) in the renal tissues. The latter reacted with the proteins of nephron, resulting in changes in the function and structure of the renal tubules and glomeruli [26].

Kidney injury molecule-1 (KIM-1) is a tubular injury marker that is widely expressed in injured renal tubular cells [27]. In the current work, the DOX group showed a significant increase in the urinary KIM-1 level when compared to the control group. This result is in agreement with the earlier report of Khames et al. (2019) [26]. These findings are supported by the histological examination in the DOX group, which showed severe interstitial hemorrhage, focal mononuclear infiltration, vascular degeneration, and cytoplasmic necrosis with pyknotic nuclei in the lining epithelium of renal tubules, as well as congested capillaries in some glomeruli. The same result was obtained in a previous study of Mahmoud et al. (2020) [28].

Interestingly, pretreatment with ADM significantly decreased serum levels of BUN, creatinine, and urinary KIM-1 and increased creatinine clearance in the ADM + DOX group. In addition, ADM significantly ameliorated the renal histological changes observed in the DOX group through the improvement of glomerular and tubulo-interstitial damage. These results supported ADM’s promising renoprotective potential in DOX-induced nephrotoxicity. Our results are consistent with the results of Oyar et al. (2012) and Wang et al. (2021) [29,30].

In the present work, the mechanisms underlying the possible renoprotective effect of ADM in DOX-induced renal injury were investigated by evaluating different oxidative stress, inflammatory, and apoptotic markers.

Our work confirmed the significant role of oxidative stress as an underlying cause of DOX-induced nephrotoxicity as indicated by an increase in MDA level, a decrease in GSH content, and a decrease in the enzyme activity of catalase. These results are supported by earlier studies of Benzer et al. (2018) and Ibrahim et al. (2020) [31,32]. A possible explanation for these results may be the one-electron reduction of DOX by several oxidoreductases to form a DOX-semiquinone radical which in turn initiates a cascade of reactions, producing ROS [31]. In addition, the oxidant metabolite of DOX has a role, as it can oxidize oxygen to give ROS, causing MDA accumulation (the degradation product of lipid peroxidation) and consumption of antioxidant enzymes [31].

Of note, these changes were suppressed by ADM co-administration. This probably occurs in part by scavenging the reactive superoxide (O2^•−^) and hydroxyl (OH^•^) radicals by ADM and blocking MDA formation from NAD(P)H oxidase and the consequent cAMP-dependent redox-sensitive gene expression, indicating reduction in lipid peroxidation and cellular injury [33].

DOX is well known to cause DNA damage via ROS-induced DNA oxidation, intercalation with DNA, and restriction of the role of the topoisomerase II enzyme during replication and transcription [30]. In this study, we used 8-OHdG, a sensitive marker of oxidative DNA damage, and DNA ladder assay. The present study demonstrated that a single intraperitoneal injection of DOX led to a significant increase in the 8-OHdG level and DNA typical band fragmentation. This result is in line with several previous reports by Benzer et al. (2018) and Afsar et al. (2020) [31,34]. Meanwhile, co-treatment with ADM decreased DNA fragmentation. Similarly, Suetomi et al. (2020) and Igarashi et al. (2014) demonstrated the cytoprotective effect of ADM in pancreatic β-cells [24], as well as acute and chronic cerebral ischemia [35]. Accumulating evidence from previous research pointed to the antioxidant mechanism of ADM, which could be accredited to activating the PI3K/Akt pathway and mitigating ER stress [36].

Furthermore, inflammation is thought to be a major cause of DOX-induced nephrotoxicity [37]. Our findings indicated a significant increase in TNF-α, IL-1β, and IL-18 levels in the DOX group; this finding is in harmony with the findings of previous research by Meng et al. (2019) and Mahmoodazdeh et al. (2020) [38,39]. This inflammatory response can be explained by oxidative stress, which can cause inflammation by redox-sensitive transcription factor activation such as NF-kB, leading to the synthesis of several pro-inflammatory cytokines [25,29]. However, ADM co-treatment effectively reduced TNF-α, IL-1β, and IL-18 levels compared to their levels in the DOX-only treated group. The ADM-observed anti-inflammatory effect against DOX-induced nephrotoxicity could be accredited to the upregulation of PPAPγ, which has been found to be involved in anti-inflammatory reactions via TNF-α downregulation due to the suppression of ER stress [36]. These findings are consistent with previous studies by Li et al. (2019) and Mahmoodazdeh et al. (2020) [21,39].

Along with its direct damaging effects, oxidative stress is important in initiating apoptosis, as excessive ROS production disrupts the mitochondrial membrane, which in turn activates the intrinsic apoptotic machinery [32]. A series of regulatory proteins regulate the mitochondrial-dependent apoptotic pathway. The pro-apoptotic Bax protein and the anti-apoptotic Bcl-2 protein are among these regulatory proteins [26,32].

The present study found that DOX increased the pro-apoptotic Bax expression, while it decreased the anti-apoptotic Bcl-2 expression, resulting in an imbalance in their ratio, ultimately promoting apoptosis. This is thought to be a reasonable response for the inflammatory and oxidative mediators that initiate and activate pro-apoptotic agents [26].

Meanwhile, the pretreatment with ADM reversed the Bax/Bcl-2 ratio. This result is in concordance with the findings Wang et al. (2021) [30] and An et al. (2017) [40]. The anti-apoptotic mechanism of ADM might be ascribed to its antioxidant, anti-inflammatory, and ER stress-attenuating effects [36]. Furthermore, Si et al. (2018) reported that ADM protected mesenchymal stem cells against hypoxia-induced apoptosis, which may be mediated through the protein kinase B (Akt)/glycogen synthase kinase (GSK)3β and Bcl-2 signaling pathways [41].

On the other hand, the NLRP3 inflammasome is a multiprotein complex that, when activated, causes caspase-1-dependent secretion of IL-1β and IL-18 as well as pyroptosis, which is an inflammatory form of cell death [42]. The ASC is required for caspase-1 recruitment and formation of an active inflammasome complex [43]. Furthermore, the linker between the amino-terminal gasdermin-N and carboxy-terminal gasdermin-C domains in GSDMD is selectively cleaved by caspase-1, which is required for the pyroptosis process to proceed [44].

According to our research, the DOX group, when compared with the control group, showed increased expression of ASC, NLRP3, caspase-1 (p20), and GSDMD-N, with an increase in the level of IL-1β and IL-18. These results are in harmony with Meng et al. (2019), who revealed that DOX may induce cardiotoxicity by enhancing the NLRP3/caspase-1-based cardiomyocyte pyroptosis [38].

DOX is thought to cause pyroptosis by increasing the expression of terminal differentiation-induced non-coding RNA (TINCR), which recruits IGF2BP and increases the expression of NLRP3, resulting in caspase-1 activation and GMDSD cleavage, in addition to IL-1β and IL-18 release [45].

We proposed that DOX-induced ROS cause the release of the NLRP3 inflammasome, which is composed of NLRP3, ASC, and procaspase-1, leading to caspase-1 activation that contributes to the maturation and secretion of IL-1 and IL18, as reported by Diao et al. (2019) [46].

On the contrary, the ADM pretreated group showed a significant decrease in ASC, NLRP3, caspase-1 (p20), and GSDMD-N expression, as well as a decrease in IL-1 and IL-18 levels. These results are in agreement with those of Li et al. (2019) [21]. The effect of ADM could be attributed to a reduction in ROS overproduction in addition to a reduction in the expression of NLRP3, ASC, caspase-1, IL-1β, and GSDMD-N, leading to slowing the progression of pyroptosis caused by DOX [21]. According to Rogers et al. (2019), GSDMD-N can also translocate and permeabilize the mitochondrial membrane, activating Bax and releasing cytochrome c, which triggers the caspase-3-mediated mitochondrial apoptotic pathway [47]. This provides a possible mechanism of ADM’s anti-apoptotic effect via a decrease in the GSDMD-N level, the executioner effector of pyroptosis, providing a link between ADM’s effect on both pyroptosis and apoptosis.

## 4. Materials and Methods

### 4.1. Animals

Twenty-eight adult male *Wistar* rats (8 weeks old) weighing 190 ± 40 g were employed in this study. Rats were obtained from Tanta University’s Research Center of Experiments and maintained in clean cages with two rats per cage at a temperature of 22 ± 2 °C and 12 h dark/light cycles. In addition, standard food and water were provided to the animals throughout the experiment. The experiment was accepted by the Medical Research Ethics Committee, Faculty of Medicine, Tanta University (No: 35005/10/21), and accomplished following the recommendations of the National Institutes of Health for the care and usage of laboratory animals (NHI Publication No. 8023, revised 1996).

### 4.2. Chemicals and Drugs

ADM, doxorubicin (as doxorubicin HCL), and other chemicals used in this study were obtained from Sigma Aldrich Co. (St. Louis, MO, USA). They were of high analytical grade. ADM was dissolved in 0.9% saline.

### 4.3. Experimental Design

Rats were acclimatized for 7 days before the beginning of the experimental protocol. Then, they were allocated into the following 4 groups (7 rats each): group I (control group), group II (DOX group), group III (ADM + DOX group), and group IV (ADM group). Rats in group I received an intraperitoneal injection (i.p) of saline (vehicle for DOX and ADM) for 14 consecutive days. Renal injury in groups II and III was induced by a single i.p injection of DOX at a dose of 15 mg/kg [48]. Moreover, rats in groups II and III received an intraperitoneal injection of saline or ADM (12 μg/kg/day) 3 days prior to and continuously for 14 days following the model being established [37]. ADM was administered to the rats in the adrenomedullin group (12 μg/kg/day i.p) [19] for 14 consecutive days (from day 1 to 14).

The human dose of DOX on the basis of body weight is 1.2–2.4 mg/kg. The doxorubicin dose in this study was selected according to the following formula, as previously described [49]:

The animal dose = Human effective dose (HED)/Km ratio.

### 4.4. Collection of Urine Samples

At the end of our study, rats were kept separately in metabolic cages for collecting 24 h urine samples. Total urine volume was estimated, and 1 mL of each sample was utilized for the determination of creatinine clearance and kidney injury molecule-1 (KIM-1) level.

### 4.5. Blood and Tissue Sampling

After collecting urine, blood samples were collected from the retro-orbital plexus 24 h later; serum was separated and stored at −80 °C until use for biochemical analysis. Following sodium pentobarbital anesthesia at a dose of 60 mg/kg i.p and cervical dislocation, animals were sacrificed. Kidneys were then cautiously dissected and rinsed with an ice-cold saline solution. For histological and immunohistochemical analysis, the left kidney from each group was held in 10% phosphate-buffered formalin. In addition, the right kidney was divided into small pieces and either homogenized as 10% (*w*/*v*) in 50 mM phosphate buffer (pH 7.4), RNA lysis buffer, or radioimmunoprecipitation assay (RIPA) buffer containing protease and phosphatase inhibitor cocktail for the determination of enzyme-linked immunosorbent assay (ELISA), real-time polymerase chain reactions (PCR), and Western blot techniques, respectively. The resultant specimens were frozen at −80 °C. Renal tissue protein level was measured by a commercial kit (Diamond Diagnostics, Egypt) using the biuret method. The remaining pieces were frozen at −80 °C until they were used to extract RNA.

### 4.6. Determination of Relative Kidney Weight

Rats’ body weights were measured at the beginning and completion of experimentation. The calculation of relative kidney weight was performed using the following formula:

(kidney weight/body weight)×100

### 4.7. Evaluation of Nephrotoxicity

Serum creatinine and blood urea nitrogen (BUN) were determined using commercial kits (Biodiagnostic, Cairo, Egypt). Creatinine clearance (an index for glomerular filtration rate) was determined using the following equation:

Creatinine clearance (mL/min) = (urine creatinine (mg/dL)×urine output (mL/24 h)/(serum creatinine (mg/dL)×1440 (min) [50]. Moreover, urinary kidney injury molecule was analyzed by an ELISA kit (Cusabio Biotech, Wuhan, China; Cat. No: CSB-E08808r) following the manufacturer’s protocol.

### 4.8. Renal Redox Status and Inflammatory Markers

Malondialdehyde (MDA) level, a marker of lipid oxidation, was quantified in renal tissues by a thiobarbituric acid method described by Ohkawa et al. [51]. Catalase activity was measured in renal tissues in accordance with the method of Sinha [52], while renal glutathione (GSH) content was assayed by the method of Beutler et al. [53].

Measurement of the levels of 8-hydroxy-2′-deoxyguanosine (8-OHdG) and TNF-α were conducted in renal tissues using ELISA kits (Sunred, Shanghai, China; Cat. No: 201-11-0032 and Cusabio Biotech, Wuhan, China; Cat. No: CSB-E11987r). The procedure was performed according to the supplier protocol.

### 4.9. Assessment of Inflammasome Activation and Pyroptosis in Renal Tissues

NLRP3 and IL-18 levels in renal tissues were assessed using commercial ELISA kits provided by Sunred Biological Technology Co., Ltd. Shanghai, China; Cat. No: 201-11-3701, 201-11-0118, and 201-11-5826, respectively, while interleukin-1 beta (IL-1β) level was measured using Invitrogen rat Elisa kits (Cat. No: KRC 0011) following the manufacturer’s protocol.

### 4.10. Determination of DNA Fragmentation by Ladder Assay

DNA extraction and fragmentation were carried out as previously explained by Suman et al., 2012 [54]. GF-1 Tissue DNA Extraction Kit, purchased from Vivantis (Shah Alam, Selangor Darul Ehsan, Malaysia), was used to isolate DNA from renal tissues. High-purity genomic DNA was eluted in water or low-salt buffers. Then, the amount of DNA was computed at 260 and 280 nm. Afterwards, 5 μL of each sample was mixed with 1 μL of loading dye (6× DNA Loading Dye, Fermentas International, Burlington, Ontario, Canada) and loaded in 1.5% agarose gel. Electrophoresis was executed at 100 V for 45 min, then DNA bands were visualized and photographed.

### 4.11. Real-Time Quantitative PCR (qPCR)

Frozen renal tissue was used to isolate total RNA by a kit protocol utilizing the Gene JET RNA purification kit (Thermo Scientific, #K0731, Waltham, MA, USA). Assessment of RNA concentration and purity were performed through assessing OD260 and OD260/280 ratio, respectively, using a NanoDrop spectrophotometer (NanoDrop Technologies, Inc. Wilmington, NC, USA). Then, 5 μg of total RNA was converted into cDNA using Revert Aid H Minus Reverse Transcriptase (Thermo Scientific, #EP0451, Waltham, MA, USA). cDNA (2 μL) was used as template for SYBR-green qPCR using Step One Plus real-time PCR system (Applied Biosystem, USA). The primer sequences were the following: *ASC* (Gene Bank Accession No. NM_172322.1)—F: (5′ GCAATGTGCTGACTGAAGGA-3′), R: (5′-TGTTCCAGGTCTGTCACCAA- 3′); *GSDMD* (Gene Bank accession No. NM_001400994.1)—F: (5′-CCAACATCTCAGGGCCCCAT-3′), R: (5′-TGGCAAGTTTCTGCCCTGGA-3′); and *β-Actin* (Gene Bank accession No. NM_031144.3)—F: (5′-GGCTGTGTTGTCCCTGTAT-3′), R: (5′-CCGCTCATTGCCGATAGTG-3′). The thermal cycling conditions were as follows: initial denaturation at 95 °C for 10 min, 40 cycles of amplification of DNA denaturation at 95 °C for 15 s, annealing at 60 °C for 30 s, extension at 72 °C for 30 s. Each reaction was performed in duplicate and the means of seven independent biological replicates were calculated. Analysis of the relative gene expression was performed using the 2^−ΔΔCt^ method [55].

### 4.12. Pyroptosis Protein Expression by Western Blotting

Protein separation (20 μg from each sample) was accomplished using sodium dodecyl sulfate (SDS)–polyacrylamide gel electrophoresis (PAGE). This was followed by the transfer of separated proteins to polyvinylidene difluoride (PVDF) membranes using Trans-Blot Turbo (Bio-Rad, CA, USA) for 7 min at 25 V. The membrane was then blocked with 3% bovine serum albumin (BSA) in 20 mM Tris Buffered Saline with 0.1% Tween (TBST). The membranes were incubated at 4 °C overnight with the following primary antibodies: anti-β-actin (1:1000, Abcam, Cambridge, UK, ab8226), anti-GSDMD-N (1:1000, Santa Cruz Biotechnology, Santa Cruz, CA, USA, sc-393,656), and anti-cleaved caspase-1 (p20) (1:1000, Gene Tex, Irvine, CA, USA, GTX11701). The images of the bands were then visualized via a CCD camera-based imager after incubation with HRP-conjugated secondary antibody and the chemiluminescent substrate (Bio-Rad, CA, USA). Finally, results were assessed after the normalization for β-actin protein expression (as the housekeeping protein) [56].

### 4.13. Immunohistochemical Analysis of the Kidney

Renal tissue expression of Bcl-2 and Bax were examined using immunohistochemical methods. Paraffin-embedded sections were dewaxed, rehydrated, and incubated with rabbit monoclonal antibodies against Bcl-2 and Bax (Dako, Carpinteria, CA, USA). Immunohistochemical photos were quantified by using image analysis software (Image J, 1.46a, NIH, Bethesda, MD, USA) [57]. Ten non-overlapping fields (400×) were examined in each slide for the mean area percentage (%) of Bax and Bcl-2 immunostaining reaction and the mean color intensity of immunohistochemical reaction.

### 4.14. Histopathological Examination of the Kidney

The left kidney from all experimental groups was preserved in 10% of neutral buffered formalin for 24 h, then embedded in paraffin wax. Finally, 4 μm thick sections were stained with hematoxylin and eosin (H&E) for histological examination by light microscopy [58]. Histological findings in the selected sections were graded on a scale from 0 to 3, as previously reported [59]. Normal histological structure is represented by 0, less than 25% damaged tissue is represented by 1, 25% to 50% damaged tissue is represented by 2, and more than 50% damaged tissue is represented by 3. The pathological parameters used in this evaluation were glomerular congestion, glomerular atrophy, degeneration of tubular epithelium, and interstitial inflammatory cell infiltration.

### 4.15. Statistical Analysis

SPSS software version 25 (SPSS Inc., Chicago, IL, USA) was used to perform the statistical analysis of our study. Data are expressed as mean ± SD. Data were tested for normality with the Shapiro–Wilk test. When *p* > 0.05, the null hypothesis was accepted, and data were normally distributed. Statistical differences between multiple groups were analyzed using one-way ANOVA followed by Tukey’s test. Kruskal–Wallis test was used to determine the difference between the groups obtained as semi-quantitative in the histopathological examination. The acceptable level of significance was at *p* < 0.05.

## 5. Conclusions

In conclusion, our findings support ADM’s potential renoprotective effects in reducing the severity of DOX-induced nephrotoxicity via the interaction of its antioxidant, anti-inflammatory, anti-apoptotic, and anti-pyroptotic properties. Similarly, pathological studies revealed that treating DOX with ADM had a noteworthy regenerative effect on the renal tissue. Based on these findings, ADM could open up new avenues for treating DOX-induced nephrotoxicity. More research is needed to confirm our outcomes.

## Figures and Tables

**Table 1:** Effect of doxorubicin and adrenomedullin treatment on body weight, relative kidney weight, serum creatinine, BUN, creatinine clearance, and urinary KIM-1 in the studied groups.

Parameter/Group	Group I (Control)	Group II (DOX)	Group III (ADM + DOX)	Group IV (ADM)
**Initial body weight (g)**	163.86 ± 8.07	165.57 ± 13.04	166.29 ± 12.19	163.43 ± 6.97
**Final body weight (g)**	190.28 ± 5.31 ^Δ^	153.29 ± 12.27 *	156.57 ± 11.86 *	188 ± 6.08 ^Δ^
**Relative kidney weight (%)**	0.29 ± 0.02 ^Δ^	0.52 ± 0.07*	0.50 ± 0.04 *	0.28 ± 0.01 ^Δ^
**Serum creatinine level (mg/dL)**	0.48 ± 0.04 ^Δ^	0.89 ± 0.02 *	0.67 ± 0.02 *^,Δ^	0.50 ± 0.03 ^Δ^
**BUN (mg/dL)**	27.51 ± 0.31 ^Δ^	42.55 ± 0.67 *	30.61 ± 0.45 *^,Δ^	27.76 ± 0.43 ^Δ^
**Creatinine clearance** **(mL/min)**	1.68 ± 0.10 ^Δ^	0.23 ± 0.04 *	0.41 ± 0.05 *^,Δ^	1.63 ± 0.09 ^Δ^
**Urinary KIM-1(ng/mL)**	6.25 ± 0.91 ^Δ^	15.40 ± 2.95 *	9.38 ± 0.90 *^,Δ^	6.79 ± 0.75 ^Δ^

Data are mean ± standard deviation. Statistical analysis was performed using one-way ANOVA with Tukey’s post hoc test, SPSS computer program. ADM: adrenomedullin; BUN: blood urea nitrogen; DOX: doxorubicin; KIM-1: kidney injury molecule-1. * Significant difference vs. control group (*p* < 0.05); ^Δ^ significant difference vs. DOX group (*p* < 0.05); n = 7 rats/each group.

**Table 2:** Effect of doxorubicin and adrenomedullin treatment on oxidative stress and inflammatory markers in rats’ renal tissues.

Parameter/Group	Group I (Control)	Group II (DOX)	Group III (ADM + DOX)	Group IV (ADM)
**Renal MDA level (nmol/mg protein)**	1.21 ± 0.23 ^Δ^	3.94 ± 0.34 *	2.35 ± 0.36 *^,Δ^	1.08 ± 0.28 ^Δ^
**Renal CAT activity** **(μmol of H_2_O_2_ consumed /min/mg protein)**	217.35 ± 6.88 ^Δ^	170.39 ± 5.27 *	200.03 ± 1.20 *^,Δ^	219.57 ± 7.48 ^Δ^
**Renal GSH level** **(μmol/mg protein)**	5.24 ± 1.01 ^Δ^	1.21 ± 0.05 *	5.06 ± 0.79 ^Δ^	4.88 ± 0.98 ^Δ^
**Renal 8-OHdG level** **(ng/mg protein)**	81.77 ± 4.78 ^Δ^	107.30 ± 2.96 *	100.53 ± 4.53 *^,Δ^	82.86 ± 4.84 ^Δ^
**Renal TNF-α level (pg/mg protein)**	13.95 ± 1.29 ^Δ^	81.75 ± 6.79 *	45.47 ± 3.49 *^,Δ^	11.86 ± 1.06 ^Δ^

Data are mean ± standard deviation. Statistical analysis was performed using one-way ANOVA with Tukey’s post hoc test, SPSS computer program. ADM: adrenomedullin; CAT: catalase; DOX: doxorubicin; MDA: malondialdehyde; GSH: reduced glutathione; 8-OHdG: 8-hydroxy-2′-deoxyguanosine; TNF-α: tumor necrosis factor α. * Significant difference vs. control group (*p* < 0.05); ^Δ^ significant difference vs. DOX group (*p* < 0.05); n = 7 rats/each group.

**Table 3:** Effect of doxorubicin and adrenomedullin treatment on inflammasome proteins and pyroptosis markers in rats’ renal tissues.

Parameter/Group	Group I (Control)	Group II (DOX)	Group III (ADM + DOX)	Group IV (ADM)
**Renal NLRP3 level** **(pg/mg protein)**	11.92 ± 0.96 ^Δ^	67.55 ± 9.53 *	28.21 ± 1.40 *^,Δ^	10.89 ± 1.07 ^Δ^
**Renal IL-1β level** **(pg/mg protein)**	42.09 ± 6.07 ^Δ^	116.69 ± 26.13 *	93.08 ± 14.47 *^,Δ^	42.50 ± 9.61 ^Δ^
**Renal IL-18 level** **(pg/mg protein)**	13.47 ± 1.93 ^Δ^	34.42 ± 4.79 *	22.13 ± 2.89 *^,Δ^	14.46 ± 1.65 ^Δ^

Data are expressed as mean ± standard deviation. Statistical analysis was performed using one-way ANOVA with Tukey’s post hoc test, SPSS computer program. ADM: adrenomedullin; DOX: doxorubicin; IL-1β: interleukin-1 beta; IL-18: interleukin-18; NLRP3: Nod-like receptor protein 3. * Significant difference vs. control group (*p* < 0.05); ^Δ^ significant difference vs. DOX group (*p* < 0.05); n = 7 rats/each group.

**Table 4:** The histopathologic score of kidney damage and morphometric analysis of Bcl2 and Bax immunostaining in the renal tissue.

Parameter/Group		Group I (Control)	Group II (DOX)	Group III (ADM + DOX)	Group IV (ADM)
**Histopathologic score**	Median	1.0	2.5	1.0	1.0
	IQR	0.00–1.0	2.0–3.0	1.0–2.0	0.00–1.0
	Mean rank	13.40 ^Δ^	33.70 *	20.10 ^Δ^	14.80 ^Δ^
**Bcl2 immune reactivity**	Mean area percentage	45.78 ± 6.58 ^Δ^	19.45 ± 6.64 *	40.59 ± 5.14 ^Δ^	45.85 ± 5.43 ^Δ^
	Color intensity	11.67 ± 1.76 ^Δ^	2.94 ± 1.82 *	10.05 ± 1.86 ^Δ^	12.08 ± 1.50 ^Δ^
**Bax** **Immune reactivity**	Mean area percentage	21.83 ± 2.55 ^Δ^	37.24 ± 4.82 *	24.11 ± 2.98 ^Δ^	19.14 ± 4.88 ^Δ^
	Color intensity	11.18 ± 1.90 ^Δ^	33.59 ± 6.11 *	13.69 ± 2.03 ^Δ^	11.54 ± 2.12 ^Δ^

Histopathological score is expressed as median and inter quartile range (IQR). Statistical analysis was performed using Kruskal–Wallis test followed by the Bonferroni-adjusted significance tests for pairwise comparison. Morphometric analyses are expressed as mean ± standard deviation. Statistical analysis was performed using one-way ANOVA with Tukey’s post hoc test, SPSS computer program. ADM: adrenomedullin; Bax: Bcl-2-associated X protein; Bcl2: B-cell lymphoma 2; DOX: doxorubicin. * Significant difference vs. control group (*p* < 0.05); ^Δ^ significant difference vs. DOX group (*p* < 0.05); n = 7 rats/each group.

## Data Availability

The data that support the findings of this study are available from the corresponding author upon reasonable request.

## Abbreviations

ADM, adrenomedullin; AKI, acute kidney injury; ASC, apoptosis-associated speck-like protein containing a caspase recruitment domain; DOX, doxorubicin; ELISA, enzyme-linked immunosorbent assay; BUN, blood urea nitrogen; GSDMD, gasdermin D; GSH, glutathione; IL, interleukin; NF-κβ, nuclear factor kappa B; 8-OHdG, 8-hydroxy-2′-deoxyguanosine; GSH, reduced glutathione; NLRP3, Nod-like receptor protein 3; ROS, reactive oxygen species; MDA, malondialdehyde; TNF-α, tumor necrosis factor-α.
